# miR-451 Loaded Exosomes Are Released by the Renal Cells in Response to Injury and Associated With Reduced Kidney Function in Human

**DOI:** 10.3389/fphys.2020.00234

**Published:** 2020-04-08

**Authors:** Manju Kumari, Aradhana Mohan, Carolyn M. Ecelbarger, Anita Saxena, Amit Gupta, Narayan Prasad, Swasti Tiwari

**Affiliations:** ^1^Department of Molecular Medicine and Biotechnology, Sanjay Gandhi Postgraduate Institute of Medical Sciences, Lucknow, India; ^2^Department of Medicine, Georgetown University, Washington, DC, United States; ^3^Department of Nephrology, Sanjay Gandhi Postgraduate Institute of Medical Sciences, Lucknow, India

**Keywords:** urinary exosomes, chronic kidney disease, diabetic kidney disease, micro-RNA, albuminuria

## Abstract

Micro-RNAs (miRs) encapsulated inside urinary exosomes (uEs) have the potential as early biomarkers. Previously, we reported that a rise in uE miR-451 predicted albuminuria in diabetic rats; however, whether the rise was protective or detrimental, and occurred in response to injury or general hyperglycemia, was unknown. To address this, we studied both human and rat models of renal disease. In humans, uE miR-451 was approximately twofold higher in subjects with early-stage chronic kidney disease (CKD; serum creatinine < 2.0 mg/dl; *n* = 28), as compared to age-matched healthy controls (*n* = 23), and had a significant negative correlation with estimated glomerular filtration rate (eGFR) (*r*^2^ = −0.10, *p* = 0.01). Subgroup analysis of CKD subjects showed that those without diabetes had slightly (∼30%) but significantly higher uE miR-451 as compared to those with diabetes, with no differences in albumin excretion, eGFR, serum sodium, and potassium. Using human proximal tubule (hPT) cells, we found that locked nucleic acid (LNA) inhibition of miR-451 resulted in a significant increase in the messenger RNA (mRNA) expression of kidney-injury-associated miR-451 targets, e.g., CAB39, TBX1, and YWHAZ, as compared to treatment with a control LNA. Moreover, hPT cells and their secreted exosomes showed an increase in miR-451 in response to mechanical injury but not high glucose (20 versus 5 mM). For further proof of concept, in diabetic rats, we showed that atorvastatin (AT), a treatment proven to attenuate renal injury without affecting systemic glucose levels, reduced uE miR-451 with the concomitant restoration of renal miR-451. These data elucidate the stimuli for renal miR-451 expression and exosomal release and support its role as a therapeutic target and early biomarker for renal injury in humans.

## Introduction

Kidney diseases impose tremendous health and economic burden on societies worldwide (Couser et al., 2011). Hypertension, diabetes, viral infection, obesity, etc., are common risk factors for kidney disease (Kazancioglu, 2013). Proteinuria, lower estimated glomerular filtration rate (eGFR), and tubulointerstitial fibrosis are linked to poor prognosis of kidney disease (Ferguson and Waikar, 2012). Early identification of kidney disease could avert the worsening of renal function. In the past few decades, urine has served as a cost-effective and non-invasive diagnostic tool to monitor kidney damage. Urine albumin/creatinine ratio (ACR) and eGFR are readily used to establish different stages of renal diseases (Koroshi, 2007; Viazzi et al., 2010). However, these parameters have some limitations regarding sensitivity and effectiveness to diagnose kidney injury (Ferguson and Waikar, 2012). This necessitates the discovery of more sensitive non-invasive biomarkers, which could improve diagnosis and aid in the early prediction of kidney diseases.

MicroRNAs being the upstream regulators of gene expression could serve as early signatures for many diseases (Cheng et al., 2005; Kajimoto et al., 2006; Bartel, 2009; Ji et al., 2009; De Guire et al., 2013). MiRNAs belong to the class of endogenous, small, non-coding RNAs, which are approximately 19–25 nucleotides long and are highly conserved across species. MiRNAs bind to 3′-untranslated region (UTR) of target messenger RNAs (mRNAs) and regulate gene expression, either by the degradation of target mRNA or translational repression (Bartel, 2004). Dysregulation of miRNAs is also reported in various renal diseases, including renal carcinoma (Jung et al., 2009), nephropathies (Trionfini and Benigni, 2017), acute and chronic kidney disease (CKD) (Saikumar et al., 2012), graft rejection (Sui et al., 2008), etc. Abundance and stability of circulating miRNAs in body fluids, such as serum, plasma, and urine, have attracted the attention of the research community to explore their role as non-invasive diagnostic biomarkers (Saikumar et al., 2014). However, unlike in the urine, the miRs encapsulated inside the urinary exosomes (uEs) are mostly of renal origin (Spanu et al., 2014). Exosomes are 30- to 100-nm intraluminal vesicles of multivesicular bodies (MVBs). These are released upon an exocytic fusion of the MVB with the plasma membrane, and their biomarker potential has been increasingly recognized (Lakkaraju and Rodriguez-Boulan, 2008). In the urine, these vesicles mostly originate from the surrounding renal cells (Pisitkun et al., 2004) and are named as “urinary exosomes.” Hence, urinary exosomal miRs may provide crucial information about renal health (Alvarez et al., 2013; Erdbrugger and Le, 2016; Lv et al., 2019). In addition, miRs encapsulated in uEs are protected from the toxic effect of the urinary environment. To this end, miRNA profiling of uEs has offered valuable insights about renal pathogenesis, and these molecules could be used for an early prediction of various kidney diseases (Chen et al., 2017; Zununi Vahed et al., 2017; Zang et al., 2019). In concordance with these studies, our lab had also reported urinary exosomal miR-451 as a signature candidate for the early prediction of diabetic nephropathy (DN) in streptozotocin-treated Wistar rats (Mohan et al., 2016).

MicroRNA-451 was first identified and reported by Altuvia et al. (2005) in the human pituitary gland more than a decade ago. The gene encoding this miRNA is located in human chromosomal region 17qll.2. It is expressed in several tissue types and participates in multiple physiological and pathological processes, including hematopoietic system differentiation, embryonic development, epithelial cell polarity, and nervous system development (Bai and Wu, 2019). Dysregulation of miR-451 in cancer and non-cancerous diseases has recently been reviewed by Khordadmehr et al. (2019). Besides its widely known dysregulation in malignancies, its overexpression in human coronary artery disease is now well appreciated (Zhang et al., 2010). With regards to kidney disease, dysregulation of miR-451 has been reported in human patients with DN and in mouse models of DN associated with mesangial hypertrophy (Wei et al., 2013; Mohan et al., 2016; Sun et al., 2016). In the previous study from our laboratory, we found a rise in miR-451 expression in uEs from diabetic rats as early as 6 weeks postdiabetes induction, and its levels continued to increase with disease progression. However, it could not be determined whether the rise in miR-451 was associated with hyperglycemia and/or kidney injury in those rats. The aim of the current study was to elucidate biomarker potential for uE miR-451 in human subjects. Specifically, we tested whether uE miR-451 levels provided valuable diagnostic information relating to stage of renal disease in both diabetic and non-diabetic subjects, i.e., its ability to detect albuminuria versus hyperglycemia. We also further explored the molecular role of miR-451 in the kidney using cell culture.

## Results

### MicroRNA 451 in Human Urinary Exosomes

**FIGURE 1:**
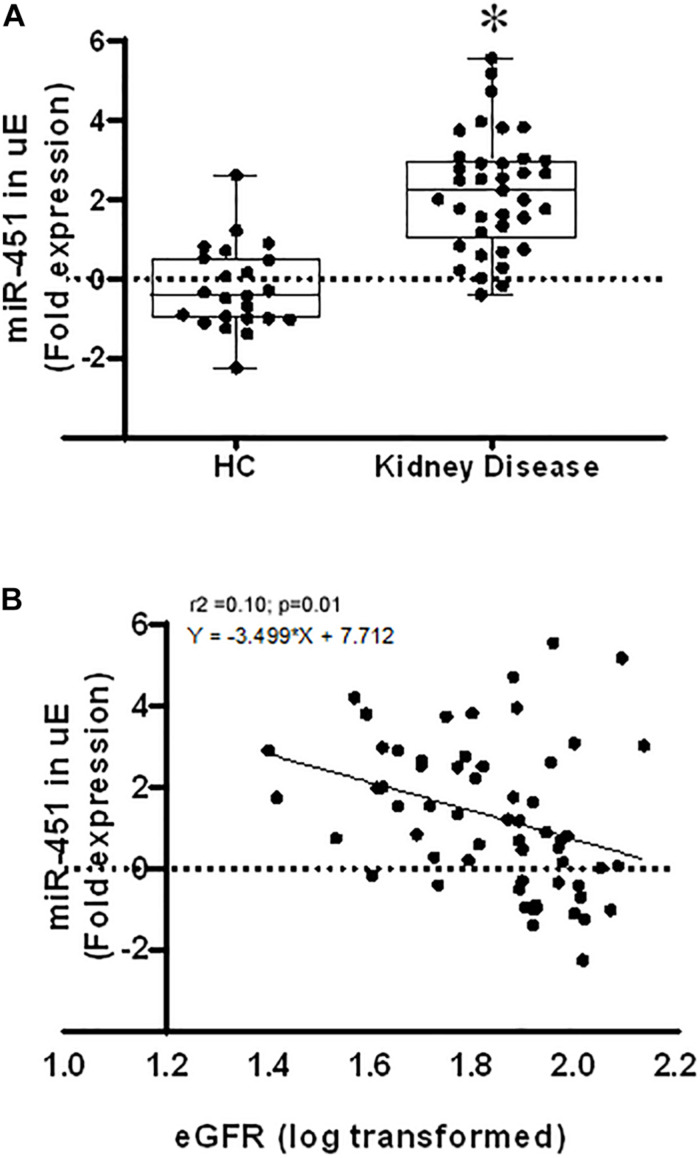
MicroRNA 451 in human urinary exosomes (uEs) and its association with kidney disease. **(A)** Box and whisker plots and scatter plot of the uEmiR-451 fold expression in healthy subjects (*n* = 23) and kidney disease patients (*n* = 38). 18S ribosomal RNA was used as control gene to calculate the fold expression of miR-451 using the formulae 2^–Δ*CT*^. Values were log transformed. Whiskers extend down to smallest value and up to largest value. *p < 0.0001 was calculated by Mann–Whitney test. **(B)** Scatter plot shows Pearson’s correlation between the uE miR-451 fold expression and estimated glomerular filtration rate (eGFR) with a best fit line curve. A significant negative correlation was found between miR-451 and eGFR.

We examined the expression of miR-451 in exosomes isolated from spot urine samples from subjects with CKD (with or without diabetes) and age-matched healthy donors. CKD subjects were found to have significantly higher uE miR-451 levels compared to healthy donors (*p* < 0.0001, Figure 1). miR-451 levels had a significant negative correlation with eGFR (Figure 1B). Table 1 describes the demographics of the enrolled subjects. The CKD subjects (*n* = 48) had increased serum creatinine, sodium, and potassium levels compared to healthy controls (*n* = 23). In addition, the eGFR and hemoglobin percentage were found markedly decreased in CKD subjects as compared to healthy controls (Table 1).

**TABLE 1:** General and physiological parameters of subjects.

Variables	Mean ± *SD*/median (IQR)		*p*-Value
	
	Controls (*n* = 23)	Kidney disease (*n* = 38)	
Urine ACR (mg/g)	3.95 (2.44−5.95)	11.69 (3.96−67.61)	0.003*
eGFR (ml/min/1.73m^2^)	93.0 (83.0−102.0)	56.0 (42.0−75.5)	0.000*
Serum creatinine (mg/dl)	1.00 (0.87−1.05)	1.50 (1.20−1.80)	0.000*
Serum sodium (mmol/L)	136 (132−140)	140 (138−143)	0.009*
Serum potassium (mmol/L)	3.98 ± 0.41	4.50 ± 0.56	0.000*
Hemoglobin (gm%)	13.3 (12.9−14.6)	12.5 (10.9−13.6)	0.002*

*Data are represented as median (interquartile range, IQR) for non-normally distributed variables and as mean ± SD for normally distributed variables. Mann–Whitney *U* test or two sample *t*-test was used as per the assumptions.*

**FIGURE 2:**
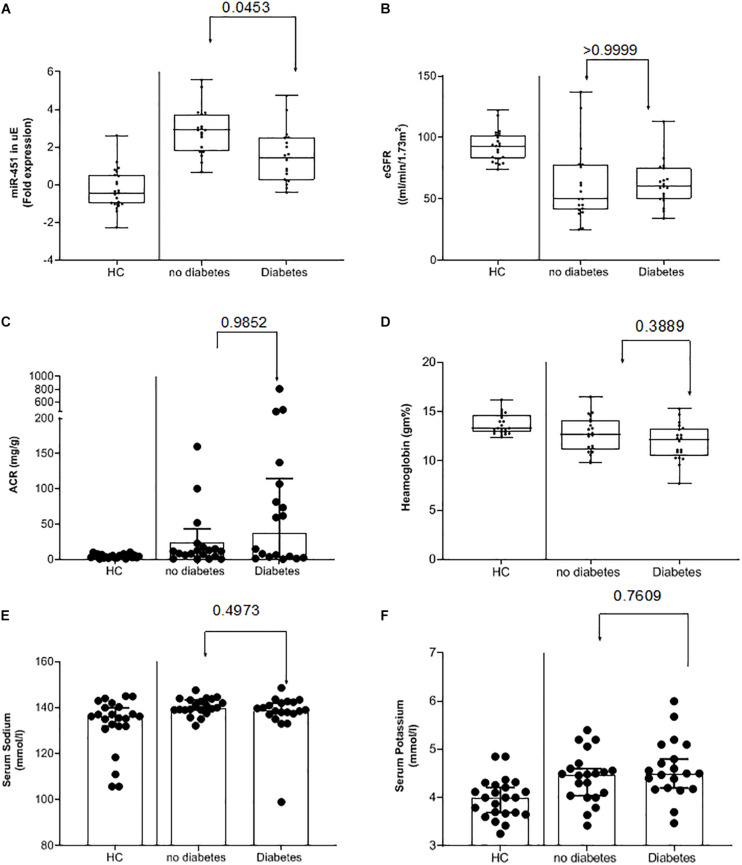
Urinary exosomal miR-451 in kidney disease subjects with or without diabetes. Box and whisker plots and scatter plot of the **(A)** urinary exosome (uE) miR-451 fold expression relative to 18S ribosomal RNA (normalizing factor) using the formulae 2^–Δ*CT*^. Values were log transformed. **(B)** Estimated glomerular filtration rate (eGFR) and **(C)** percent hemoglobin (Hb) in healthy control subjects and kidney disease subjects with or without diabetes. Whiskers extend down to smallest value and up to largest value. *P*-values were calculated by Kruskal–Wallis test. Bar graphs and scatter plot of **(D)** urine albumin/creatinine ratio (ACR), **(E)** serum sodium, and **(F)** serum potassium levels in the three groups. The median fold expression value is represented by the single line within the box with 95% CI. *p*-values were calculated by one-way ANOVA.

Subgroup analysis of the CKD subjects based on their blood glucose levels showed that CKD subjects without diabetes had significantly higher uE miR-451 expression as compared to those with diabetes (Figure 2A and Table 2A). While the other parameters, including eGFR, ACR, Hb, serum sodium, and potassium, were similar between the two CKD groups (Figure 2 and Table 2B).

**TABLE 2A:** Data are expressed as median (IQR) for non-normally distributed variables and as mean ± SD for normally distributed variables.

Variables	Mean ± SE/median(IQR)			*p*-Value
	
	Healthy controls (group 1) *n* = 23	Renal-disease subjects without diabetes (group 2) *n* = 20	Renal-disease subjects with diabetes (group 3) *n* = 18	
Urine ACR (mg/g)	4.0 (2.4–6.0)	11.7 (5.3–37.4)	37.2 (3.4–114.6)	0.005*
eGFR (ml/min/1.73m^2^)	93 (83–102)	50 (39–77)	60 (51–73)	0.000*
Serum creatinine (mg/dl)	1.0 (0.87–1.05)	1.6 (0.90–1.81)	1.5 (1.24–1.69)	0.000*
Serum sodium (mmol/L)	136 (132–140)	140 (139.00–144)	139 (137–142)	0.017*
Serum potassium (mmol/L)	4.0 ± 0.1	4.4 ± 0.1	4.6 ± 0.1	0.001*
Hemoglobin (gm%)	13.3 (12.9–14.6)	12.7 (11.1–14.1)	12.2 (10.5–13.3)	0.003*

*ANOVA or Kruskal–Wallis *H* test was used as per the assumptions.*

**TABLE 2B:** Multiple comparisons analysis using Bonferroni multiple comparison test for normally distributed variables and Mann–Whitney *U* test with correction for non-normally distributed variables.

Variables	Mean difference/*Z* statistic (*p*-value)		
	
	1 and 2	1 and 3	2 and 3
Urine ACR (mg/g)	−2.61 (0.009)	−2.49 (0.013)	−1.15 (0.258)
eGFR (ml/min/1.73m^2^)	−4.24 (0.000)	−4.87 (0.000)	−1.17 (0.240)
S. Creatinine(mg/dl)	−2.97 (0.003)	−4.64 (0.000)	−0.183 (0.855)
S. Sodium(mmol/L)	−2.645 (0.008)	−1.815 (0.070)	−1.357 (0.175)
S. Potassium(mmol/L)	−0.43 (0.020)	−0.60 (0.001)	−0.17 (0.835)
Hemoglobin (gm%)	−2.07 (0.038)	−3.363 (0.001)	−1.29 (0.196)

*p ≤ 0.016 was considered statistically significant after correction.*

### Expression and Fibrotic Targets of miR-451 in Human Renal Epithelial Cells

**FIGURE 3:**
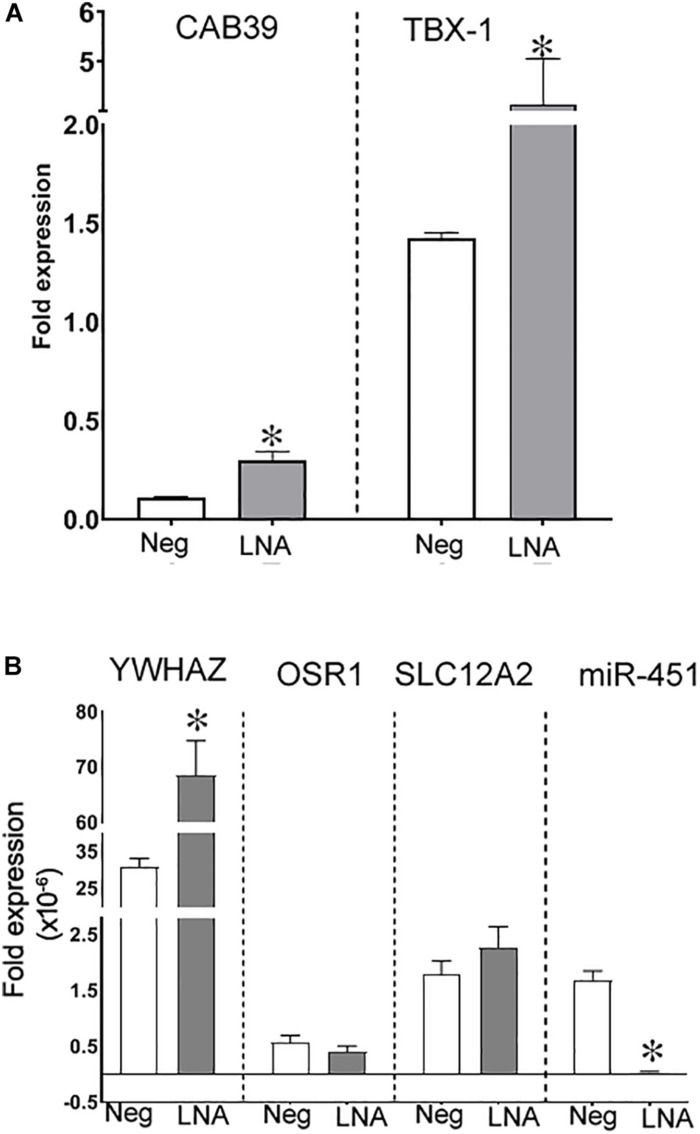
Inhibition of miR-451 in primary human proximal tubule (hPT) cells. Fold expression of **(A)** CAB39 and TBX1 and **(B)** YWHAZ, OSR1, SLC12A2, and miR-451 in hPT cells transfected either with hsa-LNA-miR-451a-inhibitor or with hsa-LNA-miR-451a-inhibitor control (negative control). Transcript levels of genes of interest were normalized against 18S ribosomal RNA using the formulae 2^–ΔC^. All values are represented as mean ± SEM. *p < 0.05 was considered significant by unpaired *t*-test.

We next determined the expression of miR-451 and its targets in human renal epithelial cells. For this, miR-451 expression was inhibited in human proximal tubule (hPT) cells using LNA-miR-451a inhibitor. Inhibition of miR-451 by locked nucleic acid (LNA) was, however, not achieved in podocytes even at the highest recommended dose of LNA. Phenotypic and functional characterization of hPT cells cultured from human kidney tissues have previously published by us (Pandey et al., 2017). The downregulation of miR-451 in hPT cells by LNA inhibitor was confirmed by real-time PCR, which showed a significant decrease in miR-451 expression as compared to LNA-miR-451a inhibitor control-transfected cells (negative control) (Figure 3). MiR-451 knockdown resulted in a significant increase in the expression of its target genes, CAB39, TBX1, and YWHAZ compared to the negative control (Figure 3). However, OSR1 and SLC12A2, other known targets of miR-451, showed comparable levels in the miR-451 knockdown and control cells (Figure 3).

Besides, in primary human podocytes, a very low abundance of miR-451 was observed. The expression of miR-451 remained unaffected in LNA-transfected cells (Supplementary Figure S1). The expression of CAB39 was also unaltered in all treatment groups (Supplementary Figure S1). The typical morphology of human podocytes, cultured from human kidney tissue, is shown in Supplementary Figure S1. Characterization of isolated human podocytes was done using podocyte-specific antipodocin and anti-WT-1 antibodies (Supplementary Figure S1). Since we found low abundance of miR-451 in podocyte and unable to see its inhibition by LNA in these cells, further experiments were conducted in hPT cells.

### Effect of Injury or High Glucose on miR-451 Expression in Human Proximal Tubule Cells

**FIGURE 4:**
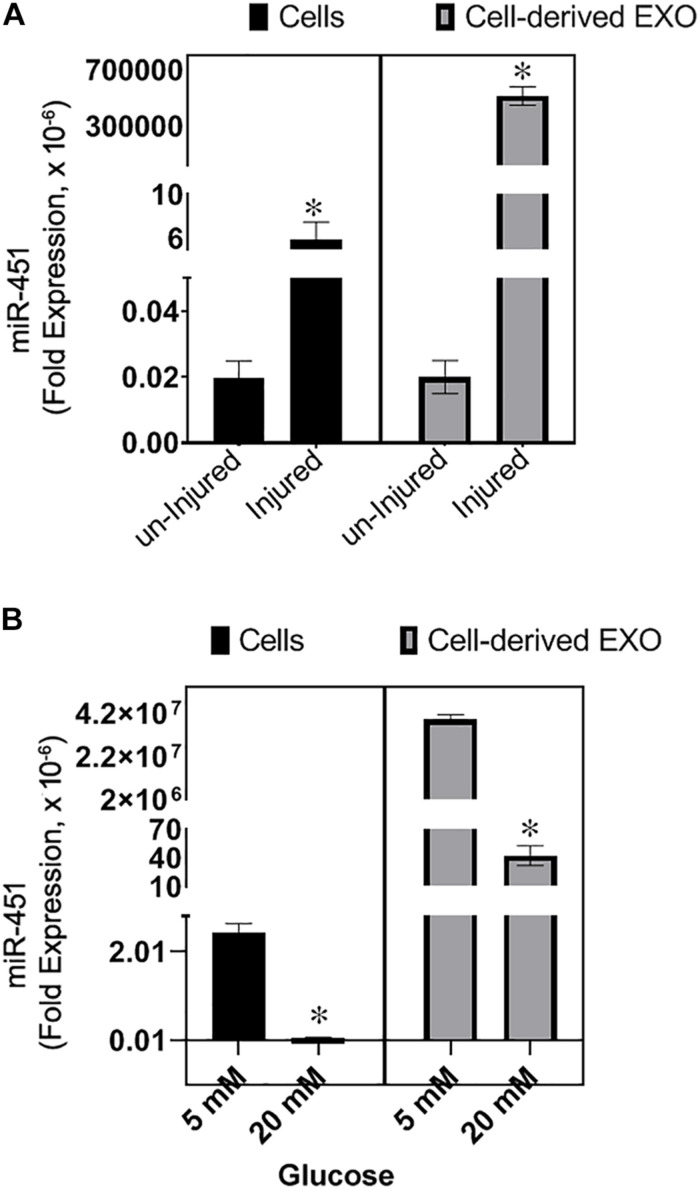
Effect of injury and high glucose on miR-451 expression in primary human proximal tubule (hPT) cells and hPT-derived exosomes. Bar graphs show fold expression of miR-451 in the hPT cells and hPT-derived exosomes in the media. **(A)** The hPT cells were injured by scratching with a 10-μl tip or remained uninjured. Total RNA was extracted after 24 h of injury. **(B)** hPT cells were cultured in the medium containing 5 or 20 mM glucose for 48 h. Fold expression of miR-451 gene was normalized against 18S ribosomal RNA using the formulae 2^–Δ*CT*^. Experiments were repeated three times. The data are represented as mean ± SEM. *p < 0.05 was considered significant by unpaired *t*-test.

To test the expression of miR-451 in response to the mechanical injury to the hPT cells such as due to cell swelling/volume changes of the cells associated with diabetes, the cells were scratched with a 10-μl tip to create injury or incubated with high glucose (20 mM). The cells and their derived exosomes were analyzed for miR-451 expression. After 24 h of injury, the cells and their secreted exosomes in the media showed an increase in the transcript levels of miR-451 (Figure 4).

**FIGURE 5:**
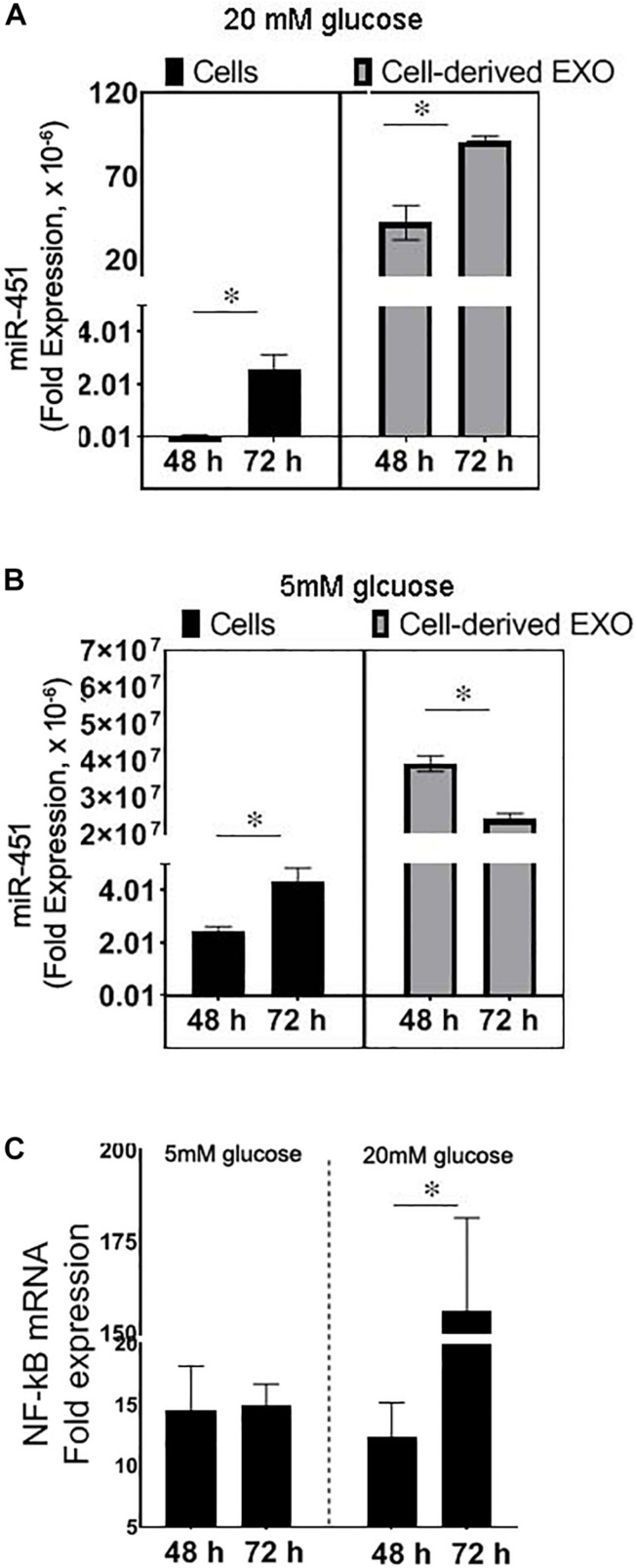
Effect of high-glucose-induced injury on miR-451 expression in primary human proximal tubule (hPT) cells and hPT-derived exosomes. Bar graphs show fold expression of **(A,B)** miR-451 in the hPT cells and hPT-derived exosomes in the media, **(C)** nuclear factor kappa-light-chain- enhancer of activated B cells (NF-κB) messenger RNA (mRNA) expression in hPT cells. Cells were cultured for 48 or 72 h in the medium containing high (20 mM) or normal glucose (5 mM). Gene expression were normalized against 18S ribosomal RNA using the formulae 2^–Δ*CT*^. Experiments were repeated three times. The data are represented as mean ± SEM. *p < 0.05 was considered significant by unpaired *t*-test.

However, in response to high glucose (20 mM), the miR-451 expression was significantly downregulated in the hPT and their secreted exosomes as compared to the cells incubated in normal glucose conditions (5 mM). The cells were incubated in high or normal glucose for 48 h (Figure 4). A longer exposure (72 h) of hPT in high glucose condition resulted in an ∼200-fold rise in miR-451 expression, relative to 48 h exposure in high glucose. Cells in normal glucose condition showed only twofold rise in miR-451 expression after 72 h relative to 48 h in culture (Figure 5A). Similarly, hPTC-derived exosomes showed a significant rise in miR-451 expression at 72 h relative to 48 h of high glucose exposure. However, under normal glucose conditions, miR-451 expression was reduced in the hPTC-derived exosomes after longer culture hours, i.e., 72 h relative to 42 h (Figure 5B). To check if the rise is associated with glucose-induced injury to the hPT cells, the expression of nuclear factor kappa-light-chain-enhancer of activated B cells (NF-κB) mRNA was analyzed. Real time reverse transcription PCR (qRT-PCR) revealed a significant rise in NF-κB mRNA levels in the hPT cells exposed to high glucose for 72 h, relative to 48 h. The expression remained similar between 48 and 72 h in the hPT cells cultured under normal glucose conditions (Figure 5C).

### Effect of Renoprotection on miR-451 Expression in Urinary Exosomes

**FIGURE 6:**
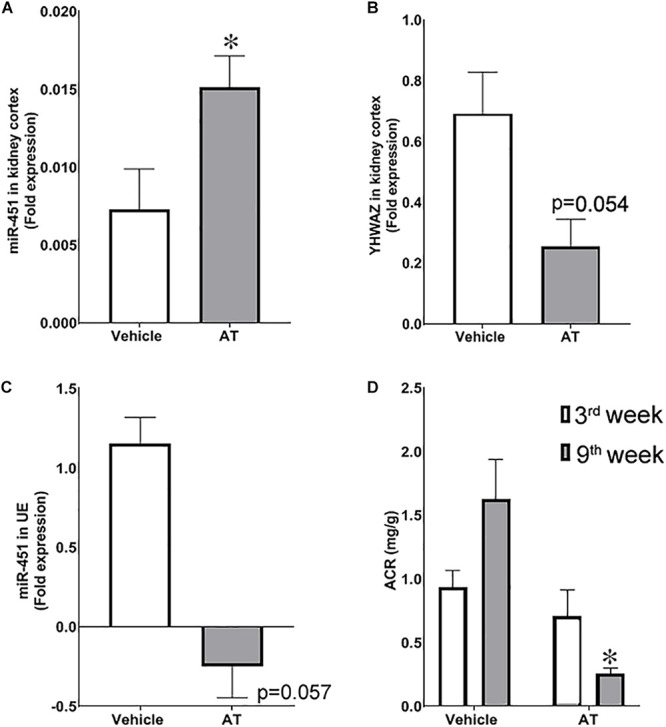
Effect of renoprotection on renal levels of miR-451 and its target, YWHAZ, in diabetic rats. Bar graphs shows fold expression of **(A)** miR-451 and **(B)** YWHAZ messenger RNA (mRNA) in the kidney cortex and **(C)** miR-451 in urinary exosomes of diabetic rats treated with atorvastatin (AT) or vehicle for 8 weeks. The 18S ribosomal RNA was used as control gene to calculate the fold expression using the formulae 2^–Δ*CT*^. **(D)** Bar graph represents urine ACR at third and eighth weeks in these rats. *p < 0.05 was considered significant by unpaired *t*-test.

We next analyzed the change in miR-451 expression in uE in response to renoprotection in rats with DN. For this, streptozotocin-treated diabetic rats were treated with AT. We have previously reported that AT (20 mg/kg body weight), a lipid-lowering agent, attenuated renal pathology in diabetic rats without affecting blood glucose levels (Singh et al., 2016). Exosomes were isolated from 8-h urine samples from diabetic rats treated with a vehicle or AT for 8 weeks. AT-treated rats were found to have lower uE miR-451 expression relative to vehicle-treated diabetic rats (*p* < 0.057, Figure 6). A significant reduction in urine ACR validated the renoprotection by AT in diabetic rats (Figure 3). Besides, renal pathology indices [tubulo-interstitial fibrosis index (TFI) and glomerulosclerosis index (GI)] were attenuated in AT-treated diabetic rats, relative to vehicle-treated diabetic rats (Singh et al., 2016).

In the kidney cortex, the miR-451 expression was significantly higher in AT-treated diabetic rats with a concomitant lower YWHAZ expression, relative to vehicle-treated rats (Figure 5). Furthermore, we observed a negative correlation between miR-451 expression and renal pathology indices [Tubulointerstitial fibrosis (TFI)/glomergular fibrosis (GI) scores] in the kidney cortex (*r*^2^ = 0.621, *p* = 0.0354 for TFI, and *r*^2^ = 0.577 and *p* = 0.048 for GI).

## Discussion

In the past few decades, urine has served as a cost-effective and non-invasive diagnostic tool to monitor kidney damage. However, the existing methods for kidney disease diagnosis, including urine ACR and eGFR have limited sensitivity and specificity. Moreover, these markers cannot be used for the early detection of kidney diseases. In our previous study, using deep sequencing of uEs, we identified miR-451-5p in uEs as potential biomarker for the early detection of renal dysfunction in an experimental model of DN (Mohan et al., 2016). In this study, we have shown higher miR-451 levels in the uE from early-stage CKD subjects and its correlation with eGFR in humans and demonstrated targets of miR-451 in human renal epithelial cells. We also provide evidence to suggest that a higher miR-451 expression in uE appears a response to cell injury but not to hyperglycemia in DN.

Our previous study suggests that a rise in miR-451 in the uEs appear earlier than any significant renal pathology and ACR increase in diabetic rats (Mohan et al., 2016). In the current study, we showed significantly higher uE miR-451 in patients with early-stage CKD compared to healthy controls. Moreover, the miR-451 expression was higher in both diabetic as well as non-diabetic CKD subjects as compared to age-matched healthy controls. Thus, alteration in miR-451 status in uE might be independent of hyperglycemia. To test this possibility, we determined the effect of renoprotection, independent of hyperglycemia, on uE miR-451 expression in rats. We have previously reported that AT attenuated renal pathology without changing blood glucose levels in diabetic rats (Singh et al., 2016). In this study, we showed that AT-treated rats had a higher miR-451 expression in the kidney cortex with a concomitant drop in uE miR-451 relative to vehicle-treated diabetic rats. Since blood glucose remained high in both AT- and vehicle-treated diabetic rats, we speculate that change in miR-451 expression was associated with renal injury and not hyperglycemia in these rats. Besides, miR-451 expression was found to increase in the exosomes secreted by the hPT cells in response to injury; however, high glucose treatment for 48 h failed to elicit this response. In response to high glucose, marked downregulation of miR-451 has been shown in mesangial cells (Sun et al., 2016; Wei et al., 2019). We also found that longer exposure of proximal tubule cells to high glucose conditions (for 72 h) led to a rise in miR451 in these cells and their derived exosomes. However, the expression remained lowered relative to cells exposed to normal glucose conditions for the same length of time. Thus, we speculate that glucose-induced injury, but not high glucose itself, is a probable cause for the miR-451 rise in renal cells and their derived exosomes. A significant rise in NF-κB transcript found in the hPT cells after 72 h of high glucose exposure, relative to 48 h, supported this hypothesis. Enhanced NF-κB has been suggested to play a dominant role in the pathogenesis of kidney injury in diabetes (Song et al., 2019). Thus, higher miR-451 levels in subjects found in our study could be associated with kidney injury.

As opposed to our observation, Sayilar and colleagues (Sayilar et al., 2016) have found significantly low urinary, but higher plasma, miR-451 in CKD patients (stages 3 and 5) as compared to healthy volunteers. The reason for this discrepancy could be the stage of renal disease at which the subjects were analyzed; our subjects were in the early-stage of CKD (1 or 2). Besides, unlike their study, we have examined uE samples instead of whole urine. Molecules in uE are thought to be protected from the toxic urine environment and may reflect true levels. In addition, biomolecules present in the urine exosomes may represent the pathophysiological state of originating renal cells, while miRs in blood filtrate may appear in the total urine in CKD subjects.

In the kidneys, proximal tubules and podocytes play major roles in renal homeostasis as well as dysfunction (Chevalier, 2016; Nagata, 2016; Waikar et al., 2016). Surprisingly, our data suggest that miR-451 may be less abundant in human podocytes. However, further investigations are warranted to draw any conclusion on the expression or role of miR-451 in human podocytes. Nevertheless, several studies have highlighted the crucial role of proximal tubules in kidney injury and dysfunction (Chevalier, 2016; Waikar et al., 2016). Being a major reabsorption site, proximal tubules are exposed to different toxic factors present in the glomerular filtrate and contribute to renal pathophysiology to a great extent (Spanu et al., 2014). Therefore, we were interested in examining the status of miR-451 in hPT cells exposed to high glucose or injury. We show that the loss of miR-451 in proximal tubules resulted in the upregulation of the YWHAZ and CAB39 genes, which might be a plausible cause of kidney fibrosis in the setting where renal miR-451 levels are low. In this line, we and others have reported an association of low renal miR-451 levels with kidney fibrosis in diabetic rats (Mohan et al., 2016; Sun et al., 2016). YWHAZ has a binding site for miR-451 in its 3′-UTR and is reported to affect renal hypertrophy via miR-451-mediated regulation of p38 mitogen-activated protein kinase (MAPK) signaling (Zhang et al., 2012). YWHAZ has a very vital role in cell proliferation (Lin et al., 2009; Nishimura et al., 2013). Thus, miR-451 may also prevent mesangial hypertrophy in DN by targeting YWHAZ (Zhang et al., 2012). Besides, miR-451 also maintains crucial renal functions and prevents renal pathology by suppressing CAB39 expression in the proximal tubules (Tian et al., 2012). Restoration of renal miR-451 levels with concomitant reduction in YWHAZ expression and attenuation of renal pathology by AT in diabetic rats further suggest a renoprotective potential of miR-451. Zhong et al. (2019) have recently reported that treatment of human proximal tubular epithelial cells with mesenchymal stem cells-derived microvesicles, which were enriched in miR-451a, reduced high-glucose or uric-acid-inflicted cell damage. They also showed that the administration of MSC-MV-miR-451a in a mouse model of DN ameliorated renal fibrosis by regulating cell cycle genes (Zhong et al., 2019). Similarly, overexpressing miR-451 expression has been shown to attenuate renal damage in db/db mice (Sun et al., 2016).

In conclusion, we have demonstrated higher uE miR-451 in subjects with early-stage CKD and provide evidence to suggest that rise in miR-451 expression in uEs could be an early response to renal cell injury. Our data also suggest that loss of renal miR-451 levels may exacerbate renal pathology.

## Materials and Methods

### Animal Study

All animal care and experimental procedures were approved by the Institutional Animal Ethics Committee (IAEC) of Sanjay Gandhi Postgraduate Institute of Medical Sciences (Registration No. 57/PO/ReBi/SL/99/CPCEA), according to the guidelines from the Committee for the Purpose of Control and Supervision on Experiments on Animals (CPCSEA), Ministry of Environment and Forests, New Delhi, India. Male Wistar rats (200–230 g) were made diabetic via single intraperitoneal administration of streptozotocin (STZ) (50 mg/kg body weight), dissolved in 0.1 M citric acid buffer (pH 4.5) after 18 h of fasting (Sigma Chemical Co., St. Louis, MO, United States), as described previously (Singh et al., 2016). Diabetic rats were fed on high cholesterol diet (DM + HC, 4% cholesterol and 1% cholic acid) (Sigma Chemical, St. Louis, MO, United States) to increase the severity of DN, as described by us previously (Singh et al., 2016). After 48 h of STZ injection, blood samples were taken from the rat tail vein, and blood glucose was monitored using a glucometer (Optimum Exceed, Abbott Diabetes Care Inc., Alameda, CA, United States). Three days after the STZ injection, these rats were either treated with AT (Cipla Ltd., Mumbai, India), an HMG-CoA reductase inhibitor (20 mg/kg body weight), or vehicle. Rats were euthanized at the end of eighth week under 2% isoflurane anesthesia (Sigma Chemical Co., St. Louis, MO, United States). Blood was collected through cardiac puncture. Kidneys were collected after perfusion with 1 × phosphate-buffered saline (PBS). Kidneys were frozen in liquid nitrogen and stored at −80°C for RNA isolation. Before euthanasia, 18-h urine samples were collected using metabolic cages (Lab Products, United States) for exosomes enrichment.

### Human Study

The study was approved by the Ethical Committee of Sanjay Gandhi Postgraduate Institute of Medical Sciences. Spot urine samples were collected from the early kidney disease subjects enrolled in the Department of Nephrology (*n* = 41). Some of these subjects had diabetes (*n* = 20). Subjects with moderate or severe kidney disease (based on their serum creatinine level > 2.0 mg/dl) were excluded from the study. Spot urine and blood samples were collected from age-matched healthy controls (*n* = 23). Spot urine was analyzed for leukocytes, blood cells, specific gravity, bilirubin, pH, ketones, uro-bilinogen, and nitrite using urine test strips. Samples were stored at −80°C for further analysis.

### Urine Albumin/Creatinine Ratio

Urine albumin was estimated using an ELISA kit (Bethyl Laboratories, TX, United States), and urine creatinine was determined by the modified Jaffe’s method using an ERBA autoanalyzer (Randox, Dublin, Ireland).

### Cell Culture Experiments

Human primary proximal tubule (hPT) cells were cultured and characterized from human kidney we previously described (Pandey et al., 2017).

#### Exposure of Renal Epithelial Cells to High Glucose or Mechanical Injury

Human proximal tubule (1 × 10^6^) cells were seeded in a six-well plate and left undisturbed until 60% confluent. Medium was then replaced with fresh culture medium containing high glucose (20 mM) or normal glucose (5.5 mM) and incubated for 48 h (Bera et al., 2017). Subsequently, culture medium was collected for exosome isolation, and cells were harvested for RNA isolation. For injury, hPT cells were seeded in six-well plates, and a sterile 10-μl tip was used to scratch the cells, as described earlier (Yung and Davies, 1998). After 24 h, the cells and their media-derived exosomes were collected for total RNA isolation.

#### MiR-451 Inhibition in Renal Epithelial Cells

Human proximal tubule cells (5 × 10^4^/well) were cultured in 12-well plates under normal culture conditions [5% CO_2_ and 95% O_2_ in Dulbecco’s modified Eagle’s medium (DMEM)/F12 medium with supplements]. For RNA interference experiments, 60–70% confluent cells were transfected with miRCURY LNA, a miRNA inhibitor against human miR-451a (HSA-miR-451a inhibitor, 50 nM) and miRCURY LNA negative, a miRNA inhibitor control (HSA-miR-451a negative control, 50 nM) as per the manufacturer’s instructions (Qiagen, Hilden, Germany). Briefly, Lipofectamine RNAiMAX transfection reagent (Invitrogen, United States) was used for transfection. After 48 h of transfection, the cells were harvested for total RNA isolation and real-time quantitative PCR (qPCR).

### Urinary Exosomes Isolation and Characterization

Urinary exosomes were isolated from rat and human urine samples by ultracentrifugation and characterized as previously described by us (Kalani et al., 2013; Mohan et al., 2016).

### RNA Isolation From uE and Kidney Tissue

Total RNA was extracted from the urinary exosomal pellets using miRNeasy Mini kit (Qiagen, Valencia, CA, United States) as per the manufacturer’s instructions. Total RNA from rat kidney was extracted using RiboZol reagent (Amresco OH, United States).

### Real-Time qPCR

To measure microRNA-451 expression, complementary DNA (cDNA) was prepared using 10 ng of total RNA, enriched from uEs, as well as from the kidney tissues using commercially available Taqman microRNA expression assays (miR-451a assay by Applied BioSystems, Foster City, CA, United States) as per the manufacturer’s instructions. qPCR was performed using Applied Biosystems 7900HT Fast Real-Time PCR system. Data were analyzed by the 2^–Δ*Ct*^ method, using 18S ribosomal RNA (rRNA) as an endogenous control.

To measure expression of miR-451 targets, total RNA (10 μg) from kidney tissues was reverse transcribed using High-Capacity cDNA Reverse Transcription Kit (Applied Bio Systems, Foster City, CA, United States) as per the manufacturer’s instructions using random primers. qPCR analysis for relative quantification of target genes of miR-451, such as CAB39, YWHAZ, OSR1, SLC12A2, and TBX1 in the kidney tissues were performed using SYBR Green Master Mix probes (TaKaRa, Kusatsu, Japan) as per the manufacturer’s instructions. Samples were loaded onto 96-well plate format, and qPCR was performed using a 7900HT Fast Real-Time PCR System. The relative gene expression was analyzed using the 2^–ΔΔ*Ct*^ method with 18S rRNA used as an endogenous control. The primers sequences are MiR-451a (AAACCGUUACCAUUACUGAGUU), CAB39 (forward: 5′-CACGTTTTTAAGGTGTTTGTA-3′, reverse: 5′-A TCCTCCGTCCTGTCGTTCTG-3′); OSR1 (forward: 5′-TGAGC GACCTTACACCTGTGACAT-3′, reverse: 5′-TGGCAGAATCC TTTCCCACACTCT-3′); SLC12A2 (forward: 5′-CAAGACATA CCGGCAGATCAG-3′, reverse: 5′-ACTAGACACAGCACCTTT TCG-3′); TBX1 (forward: 5′-ACAACCTACTGGACGACAACG G-3′, reverse: 5′-CGGCATATTTCTCGCTATCTTT-3′); YWH AZ (forward: F: 5′-TGATCCCCAATGCTTCACAAG-3′, reverse: 5′-GCCAAGTAACGGTAGTAATCTCC-3′); and NF-κB (forward 5′-GAAGCACGAATGACAGAGGC-3′, reverse 5′-G CTTGGCGGATTAGCTCTTTT-3′).

### Statistical Analysis

The normality of the continuous data was tested using the Shapiro–Wilk test. Normally distributed data from human studies were expressed using means ± SD. Data that were not distributed normally were expressed as median [interquartile range (IQR)]. The Mann–Whitney *U* test was used to compare two different parameters. Kruskal–Wallis *H* test was used for comparison among more than two groups. A *p* < 0.05 was considered as significant with the two-tailed alternate hypothesis. All the statistical analysis was done using Sigma plot 12.3 (Chicago, IL, United States) or GraphPad software (San Diego, CA, United States) and tables were drawn using SPSS software (IBM, New York, NY, United States).

## Data Availability Statement

All datasets generated for this study are included in the article/Supplementary Material.

## Ethics Statement

All animal care and experimental procedures were approved by the Institutional Animal Ethics Committee (IAEC) of Sanjay Gandhi Postgraduate Institute of Medical Sciences (Registration No. 57/PO/ReBi/SL/99/CPCEA), according to the guidelines from the Committee for the Purpose of Control and Supervision on Experiments on Animals (CPCSEA), Ministry of Environment and Forests, New Delhi, India. The study involving human subjects was approved by the Ethical Committee of Sanjay Gandhi Postgraduate Institute of Medical Sciences.

## Author Contributions

ST, AG, NP, and AS contributed to the data research idea and the study design. MK and AM contributed to the data acquisition. ST, MK, AG, and NP contributed to the data analysis and interpretation. ST, CE, and MK contributed to the important intellectual content during manuscript drafting. Each author have read and approved the final version of the manuscript, accepted personal accountability for the author’s own contributions, and agreed to ensure that questions pertaining to the accuracy or integrity of any portion of the work are appropriately investigated and resolved.

## Conflict of Interest

The authors declare that the research was conducted in the absence of any commercial or financial relationships that could be construed as a potential conflict of interest.

## Supplementary Material

FIGURE S1 MicroRNA 451 in human podocytes. **(A)** Figure shows representative immunofluorescence images of primary podocytes, isolated from human kidney. The cells were stained for podocyte-specific proteins, podocin and Wilm’s tumor (WT1). **(B)** Figure shows fold expression of miR-451 and CAB39 (target of miR-451) in primary human podocytes transfected with hsa-LNA-miR-451a-inhibitor or hsa-LNA-miR-451a-inhibitor control (negative control). The 18S ribosomal RNA was used as control gene to calculate the fold expression using the formulae 2^–Δ*CT*^. The data are represented as mean ± SEM. *P* < 0.05 was considered significant by unpaired *t*-test.
