# Corrigendum: miR-451 Loaded Exosomes Are Released by the Renal Cells in Response to Injury and Associated With Reduced Kidney Function in Human

**DOI:** 10.3389/fphys.2020.00844

**Published:** 2020-07-28

**Authors:** Manju Kumari, Aradhana Mohan, Carolyn M. Ecelbarger, Anita Saxena, Amit Gupta, Narayan Prasad, Swasti Tiwari

**Affiliations:** ^1^Department of Molecular Medicine and Biotechnology, Sanjay Gandhi Postgraduate Institute of Medical Sciences, Lucknow, India; ^2^Department of Medicine, Georgetown University, Washington, DC, United States; ^3^Department of Nephrology, Sanjay Gandhi Postgraduate Institute of Medical Sciences, Lucknow, India

**Keywords:** urinary exosomes, chronic kidney disease, diabetic kidney disease, micro-RNA, albuminuria

**Anita Saxena** was not included as an author in the published article. The corrected Author contributions statement appears below.

The authors apologize for these errors and state that they do not change the scientific conclusions of the article in any way. The original article has been updated.

## Author Contributions

ST, AG, NP, and AS research idea and study design. MK and AM data acquisition. ST, MK, AG, and NP data analysis/interpretation. ST, CE, and MK important intellectual content during manuscript drafting. Each author have read and approved the final version of the manuscript, accepted personal accountability for the author's own contributions, and agrees to ensure that questions pertaining to the accuracy or integrity of any portion of the work are appropriately investigated and resolved.

