# Supplementary figures and images for: miR-451 Loaded Exosomes Are Released by the Renal Cells in Response to Injury and Associated With Reduced Kidney Function in Human
Source: Front Physiol. 2020 Apr 8;11:234. doi: 10.3389/fphys.2020.00234 (PMC7158952; doi:10.3389/fphys.2020.00234)

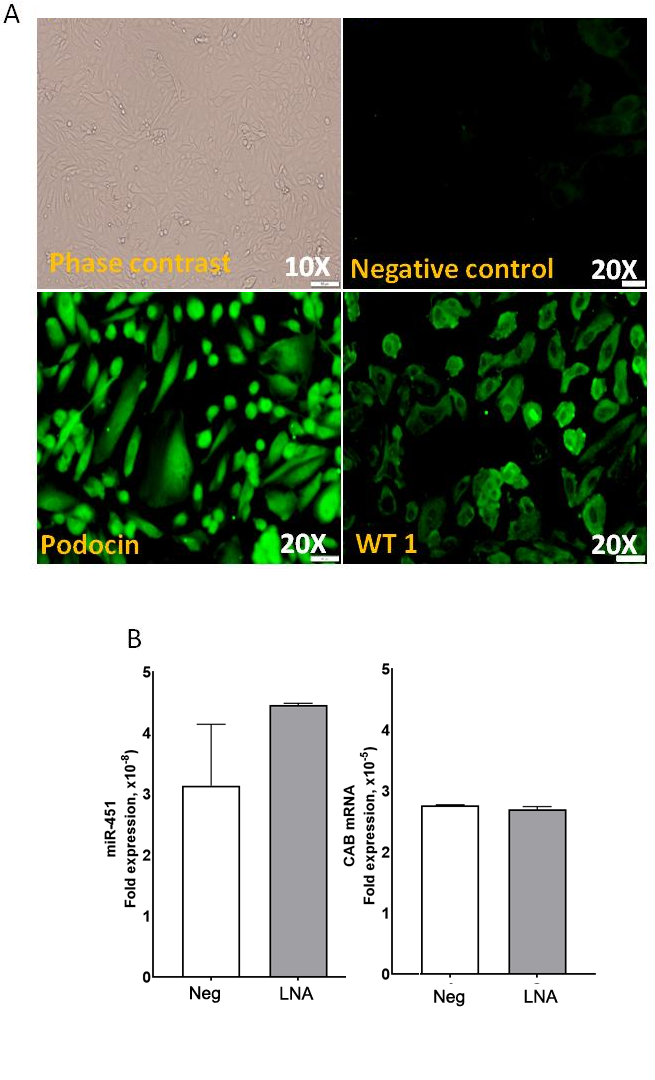

Supplement: FIGURE S1 — MicroRNA 451 in human podocytes. (A) Figure shows representative immunofluorescence images of primary podocytes, isolated from human kidney. The cells were stained for podocyte-specific proteins, podocin and Wilm’s tumor (WT1). (B) Figure shows fold expression of miR-451 and CAB39 (target of miR-451) in primary human podocytes transfected with hsa-LNA-miR-451a-inhibitor or hsa-LNA-miR-451a-inhibitor control (negative control). The 18S ribosomal RNA was used as control gene to calculate the fold expression using the formulae 2–ΔCT. The data are represented as mean ± SEM. P < 0.05 was considered significant by unpaired t-test. [file Image_1.TIF]
